# Beyond platform capitalism: critical perspectives on Facebook markets from Melanesia

**DOI:** 10.1177/01634437211022714

**Published:** 2021-06-09

**Authors:** Geoffrey Hobbis, Stephanie Ketterer Hobbis

**Affiliations:** University of Groningen, The Netherlands; Wageningen University, The Netherlands

**Keywords:** buy and sell, Facebook, informal markets, non-capitalist societies, platform capitalism, Solomon Islands

## Abstract

This article argues for a need to move beyond studies of platform capitalism and inter-capitalist struggles to also account for inter-economic struggles, the platformization of longstanding primarily non-capitalist societies, the same kind of societies that have conceptually inspired discussions of platforms as hi-tech gift economies. Based on longitudinal ethnographic fieldwork on digital transformations among the horticulturalist Lau of Malaita, Solomon Islands, we analyse horticulturalist adoptions and adaptations of Facebook. Specifically, we consider how informal bush markets are being digitized through online Buy and Sell groups. We show how Solomon Islanders use Buy and Sell Facebook groups to continue moral economic practices that emphasize the accumulation of wealth not in a capitalist, but in a relational sense, where economic activity primarily serves the creation and affirmation of relationships. Our findings, thus, challenge universalizing claims about the nature of platforms as one that is necessarily about the commodification, in a capitalist sense, of all social relations. Simultaneously, they call for more research on experiences of platformization at the margins of global capitalism and the ways in which not-so-average users are making platforms their own.

## Introduction

In this article we argue that the current vogue of studying ‘platform capitalism’ (Srnicek, 2017), in essence the commodification of web 2.0, has failed to generate a genuinely global perspective on digital economic transformations. Platform studies increasingly postulates that the global proliferation of digital platforms, such as Facebook or Google, operates similarly to colonization processes just on a more insidious, individual, personalized level (Couldry and Mejias, 2019). By virtue of their designs and the business interests that inform them, platforms are said to extract and commodify resources, in this case personal user data, where ever they go. Therefore, they are argued to spread capitalist needs and values into every corner of the globe. We contend that this perspective is essentially trapped in a ‘capitalist realism’ (Fisher, 2009) that is unable to think beyond capitalist presences, ignoring contemporary, longstanding *other* economic systems of production, distribution and consumption such as hunter-gatherers, horticulturalists, pastoralists or non-industrial agriculturalists. These *other* economies have long encountered, resisted and proven resilient in the face of capitalist colonization. It stands to reason that they may do the same in response to a now digital capitalist colonialism.

Based on longitudinal ethnographic research on digital transformations in primarily horticultural Solomon Islands and specifically an investigation into Buy and Sell Groups on Facebook, this article demonstrates this very resilience. We detail the emergence of a ‘platform horticulturalism’ where, in our case, Facebook encounters, transforms and is transformed by an economic system dominated by sustainable, rainforest-based gardening and fishing and networks of reciprocal exchange at ‘informal’ offline, bush and online, Facebook markets. We, therefore, call for a perspectival shift in how platforms are studied today: from a primary emphasis on capitalist expansion and ‘inter-capitalist struggles’ (Prey, 2020: 8) to a consideration of *inter-economic* struggles.

## Solomon Islands

People do not typically look to Solomon Islands for digital insights. The first place in the Pacific to be ‘discovered’ by Europeans in 1568, this archipelago was one of the last regions to be formally colonized. Largely due to its limited promise as a site for intensive resource extraction in comparison to the costs associated with colonization, it only became a protectorate in the then British Empire in 1893. Gaining political independence in 1978, Solomon Islands today is among the most aid dependent countries in the world (World Bank, 2018) rendering it effectively within the neocolonial soft power influence of global donors, especially its immediate neighbours Australia and New Zealand but also the United States and increasingly China. In addition, since independence the country’s Westminster, first-past-the-post system has struggled to be reconciled with customary systems, small-scale political units centred around communally owned land and claims to leadership that are largely contingent on reciprocal redistributions of wealth, for example, through contributions to community feasts (Hobbis and Hobbis, 2021). Between 1998 and 2003 these political tensions, combined with global dependencies, contributed to a civil conflict, lead to Solomon Islands international classification as ‘failed state’ and, between 2003 and 2017, the presence of an Australia-led regional military and civil assistance mission. Post-intervention, the long-term stability of the Solomon Islands state remains in question, as core root causes of the conflict have yet to been addressed (Moore, 2018).

Still, Solomon Islands population of less than 700,000 has been incredibly resilient. Its geography might be key. An archipelago of over 900 islands strung northwest to southeast in the tropic of Capricorn, these islands are covered in lush jungle and surrounded by oceans teaming with life, supplying most of the materials used on a daily basis for socio-economic reproduction, especially foods and housing. In this environment a robust, though by global environmental changes increasingly challenged, contemporary yet historically longstanding horticultural economy dominates. Nearly 80% of its population live in rural environments on the extreme frontier of capitalism. Daily life for most Solomon Islanders is typified by gardening and fishing and, to make up for gaps and surpluses in these activities, the reciprocal gifting of produce within social networks and occasional exchange or sale of them at ‘informal’ bush markets.

When life becomes more difficult is when Solomon Islanders seek to access the cash economy beyond these bush markets, primarily to afford particular ‘modern’ services such as schools and health care facilities or foreign produced goods such as the bush knives or machetes needed for garden work or more recently mobile phones. Access to the cash economy is usually limited to temporary internal labour migration, especially to the capital, Honiara on the neighbouring island of Guadalcanal. Urban residents have more regular access to the ‘formal’ economy, including salaried positions, but often struggle to afford, for example, regular access to electricity and other grid-based infrastructures. Simultaneously, infrastructures themselves are limited. For example, digital technologies face a lack of pre-existing cable infrastructures. The vast majority of network connections are facilitated through broadcast over airwaves which struggle to get into the nooks and crannies of mountainous jungle and terrain, being further frustrated by the fact that signal strength dissipates over water (see Hobbis and Hobbis, 2020).

Despite such constraints, digitization has been prosaic and radical in the country. While consumer statistics for digital technologies in Solomon Islands are unreliable, for example, by not accounting for shared mobile phone use (see Hobbis, 2019), they do paint a picture of the speed at which digital technologies have proliferated despite these constraints: Between 2010 and 2018 mobile phone penetration jumped from 20% to 80% (Telecommunication Commission Solomon Islands [TCSI], 2020). Facebook records approximately 122,000 members as of October 2020, nearly 20% of the population (Kekea, 2020) and comparable to the amount of Internet users more broadly (TCSI, 2020). A confluence of forces has facilitated the accessibility of mobile phones, which are also primary Internet access points. Most significantly, in 2010, the monopoly of the state-owned OurTelekom was dissolved, and a competitor, PNG-based Bmobile, entered the market. Prices were brought down and a race to install telecommunications infrastructures across Solomon Islands provinces began.

As direct access to the Internet spreads through the country, more and more Solomon Islanders are investing the little money they have into spending time online, mainly Facebook. This increasing popularity of Facebook represents a cause celebre in the lives of Solomon Islanders: Facebook has catapulted political tensions online, unmasking, for example, cases of corruption and sharing evidence of infrastructural decay from across the country; while also spreading accusations of sorcery and witchcraft and being used for political machinations and smear campaigns. Solomon Islands Facebook, thus, echoes much of the broader debates about social media platforms. As it has done in other places, Facebook promotes a culture of sharability as well as openeness and transparency (e.g. see Bertot et al., 2010; Li et al., 2020) while providing much fuel for disagreement, contestation and even violence (e.g. see Ganesh, 2018; Karekwaivanane, 2019). However, we contend that the story about platforms in Solomon Islands, in this case specifically Facebook, is not that simple and that understanding the platformization of Solomon Islands offers new insights into the ways social media platforms have become part of lifeworlds around the world, including societies at the margins of global capital.

The fact that our case study is based off of a society with a largely horticulturalist rather than capitalist economy is no small matter. The vast majority of interdisciplinary research on platforms such as Facebook including ethnographic work has been done in capitalist, (post)industrial societies. This is exemplified in the Why We Post Project (Miller et al., 2016) which sought to compare global uses and consequences of social media through ethnographic research, from low-income communities in Brazil to an English village. Project members did, however, never leave the discursive straightjacket of capitalist economies; to put it simply, they never went beyond the kind of place where one can find a McDonalds whether it be one that serves Mutton Burgers as in urban India or Halal McMeals in Turkey. Solomon Islands is not one of those places and, therefore, as we argue in the following, opens up new ways to think through and about platforms, in particular, the extent to which they and their users are necessarily trapped within capitalist socio-economic relations.

## Platform studies today

People encounter platforms when they log onto the increasingly familiar landscape of Facebook, Google, Amazon or Spotify, where the rapid commercialization of user-generated content and participatory online cultures, ‘Web 2.0’, has transformed social realities at whiplash speed. A new generation of academics has scrambled to get a grip on the rapid evolution and distribution of this new species of digital life (e.g. see Montfort and Bogost, 2009; Plantin et al., 2018; Srnicek, 2017; van Dijck, 2013). These scholars seek to understand how social contexts are shaped by, and shape, platforms as ‘a programmable architecture designed to organize interactions between users’ and ‘fuelled by data, automated and organized through algorithms and interfaces, formalized through ownership relations driven by business models, and governed through user agreements’ (van Dijck et al., 2018: 9; emphasis removed).

Because of their programmability and their emphasis on two-way communication, platforms were initially praised as offering alternatives to dominant political and economic systems, for example, by replacing university-based education through ‘free’ platforms such as Corsera. However, interdisciplinary platform scholars’ perspectives have quickly become more critical. Despite their promises, platforms have revealed themselves as, first and foremost, ‘economic actors within a capitalist mode of production’ (Srnicek, 2017: 3; see also Langley and Leyshon, 2017). Rather than realizing a possibly more equal ‘sharing economy’ through their very design, platforms seem to perform ‘the structure of the venture capital investment which also backs it’ (Langley and Leyshon, 2017: 3). For example, the ‘share’ or ‘like’ buttons standard on many platforms may allow users to express themselves but they also, and above all, ‘facilitate ranking, product recommendations and data analytics’ (Plantin et al., 2018: 297). Their goal is to have more, and better, data to extract as a resource to be sold in ways that are not that different from ‘the predatory extractive practices of historical colonialism’ (Couldry and Mejias, 2019: 337). Similarly, rather than serving as space where users can freely generate and share content, platforms actively curate their content through features such as the playlists on Spotify (Prey, 2020) or computational processing techniques that variously recompose digital images ‘[reifying] certain representations while discriminating against others’ (Taffel, 2021: 250). Commercial interests essentially drive this curation ‘[inaugurating] relations of dependency among creators and the industries they draw upon’ (Prey, 2020: 1; see also Towse, 2020).

Having identified this tension between ‘platform capitalism’ (Srnicek, 2017) and the participatory promises of platforms, platform studies today largely revolves around analyses of powerplays between platforms, markets and the broader ecosystems in which they are embedded. Following what is essentially a sociotechnical approach to the digital (see Hobbis, 2020; Nova, 2018), platform scholars are, in particular, exploring ‘tensions between agency and architecture’ (Plantin et al., 2018: 297), accounting for the agency of platforms themselves, as much as for the agency of creative industries, state actors and consumers (e.g. see Lin and de Kloet, 2019; Nieborg and Poell, 2018; Prey, 2020). These tensions have revealed platforms as a ‘set of relations that constantly need to be performed’ (van Dijck, 2013: 26) and that give actors other than platforms some space to manoeuvre, allowing, for example, undereducated, marginalized Chinese youth to find success as ‘self-employed “creative workers”’ (Lin and de Kloet, 2019: 1). Platforms may be designed to capture other actors, roping them further into the capitalist system that feeds and is fed by them (Langley and Leyshon, 2017; Srnicek, 2017). Still, these other actors are not, in van Dijck et al.’s (2018) words ‘“puppets” of the techno-commercial dynamics inscribed in a platform’ (p. 11). Instead, the power of platforms is ‘an always unstable and shifting outcome of the ongoing attempt to coordinate between these markets and actors’, revealing ‘inter-capitalist struggles’ (Prey, 2020: 8).

Platform Studies, thus, recognizes the messiness inherent in encounters between platforms, markets, governments and individual users. Nonetheless, current research has insisted on following a particular path to uncovering these struggles and the power imbalances embedded therein. Above all, platform scholars have primarily followed Montfort and Bogost’s (2009) map. Montfort and Bogost (2009) ask researchers to prioritize analyses of ‘a single platform or a closely related family of platforms’, based on ‘technical rigor and in-depth investigation of how computing technologies work’, and awareness and discussion of ‘how computing platforms exist in a context of culture and society’ (p. vii). In other words, their map proposes that to understand platforms scholars have to move beyond end-users and focus, instead, on the rest of ‘the much bigger, invisible technical dimensions of the platform, as well as its business dimension, which together dictate how the platform is transforming over time and how it interacts with other inhabitants in the wider ecosystem’ (Nieborg and Helmond, 2019: 198).

Guided by these propositions, scholars interested in unlocking the nature of platforms have engaged mainly with them through their designers and programmers. The focus has been on ‘paratexts’ such as marketing materials (Apperley and Parikka, 2018) and on network, discourse and content analyses of APIs. In other words, despite a recognition that platforms are always contested by highlighting their technical dimensions, platform studies today has primarily searched for what is ‘stable’ and ‘consistent’ in the ‘material-discursive conditions’ (Apperley and Parikka, 2018: 53) that surround particular platforms. It has ‘often taken a top-down perspective, disregarding bottom-up processes of (re-appropriation)’ (Werner, 2017: 183) and, thus, created perceptions of platforms as ‘uniform’ (Apperley and Parikka, 2018: 353) with ‘average’ users (e.g. see van Dijck, 2013) that are unable to escape the capitalist groundings of platform designs. From this perspective platforms then seemingly necessarily (‘commodify’) *all* social relations by collecting, algorithmically processing, circulating, and selling user data’ (de Kloet et al., 2019: 249; emphasis added) irrespective of the extent to which other actors may be able to identify a room to manoeuvre within them.

## An anthropological intervention

In February 2014, we joined the ranks of this new and flourishing field when we arrived in Solomon Islands. We stayed for a year to use classically conceived Oceanic ethnography to understand the cutting edge of digital transformations and Solomon Islands integration into the global political economy. We conducted a multi-sited ethnography travelling specifically with the Lau-speakers of Malaita Province between Honiara’s squatter settlements to their ancestral home in the rural Lau Lagoon; and occupied our time in a marathon of participant observation and documentation. To adapt a quote from Lewis (1998), we essentially tried to live as the Lau live and most of all with them, ‘to learn about their feelings, values, problems, the bases of their social relations, their economic struggles and political travails; to seek to understand their rituals and beliefs’ (p. 721), as a way to get closer to them, much more so than for example, merely studying Lau presences on Facebook would have allowed us to do.

In addition, after 8 months of rapport building and context construction, we deployed an object-centric, semi-structured sociocultural smartphone research protocol that builds on previous anthropological research on mobile phones by, among others, Horst and Miller (2006). We sat down with handset owners and discussed everything from the hardware to the software, ranging from object biographies to the contents of SIM cards, which showed communicative networks, and MicroSD cards, which showed media networks. We conducted a total of 100 interviews with close to gender parity from respondents. And, we complimented the smartphone protocol we a series of targeted interviews designed to understand better the broader material ecology, such as on road and telecommunication infrastructures. Finally, we have spent much of the past decade observing and participating in Solomon Islands Facebook, joining the many Solomon Islands-focused groups and observing discussions taking place on them, while situating these observations in our broader ethnography of Solomon Islands. Thus, we studied, in as far as possible holistically, first encounters with the Internet that has in Solomon Islands never been anything but its iteration in Web 2.0.

What makes Solomon Islands so interesting for Platform Studies is that much of its theoretical basis engages with the participatory potentials of platforms has come from even earlier ethnographic studies of primarily gift rather than capitalist economies. Exemplary is Barbrook’s (1998) discussion of a ‘hi-tech gift economy’ where ‘the dotcom commodity economy’ and gift relations ‘are—at one and the same time—in opposition and in symbiosis with each other’ (see also Elder-Vass, 2016; Fourcade and Kluttz, 2020). Platform Studies has, for this purpose, engaged with famous cases such as the Kula Ring, an Island Melanesian reciprocal system of exchange that values not individual accumulations of wealth but acts of giving and receiving as a source of fame and fortune, as well as the similarly reciprocity-centric potlach practising societies of Vancouver Island in the northern Pacific region of Canada and associated areas such as the Kwakwaka’wakw, Nuu-chah-nulth and Coast Salish Peoples. These are, arguably, the original participatory cultures typified by extreme egalitarian social relationship predicated not primarily on individual conceptualizations of personhood but on one that is dividual, wherein ‘persons are frequently constructed as the plural and composite site of the relationships that produce them’ (Strathern, 1988: 285). It is such participatory cultures that informed Mauss’ (1990 [1925]) landmark monograph *The Gift* and, thus, contributed significantly to Western discourses on reciprocity and sharing as a response to unfettered capitalist extraction, to their attempted realizations in alternative forms of living, such as ecovillages, in alternative perspectives on economic relations, such the ‘sharing economy’, or in alternative media practices such as reciprocal liking on Facebook (see Fourcade and Kluttz, 2020).

When engaging with these societies Platform Studies has, however, often only interrogated dated ethnographic texts, or even more so earlier theoretical reflections on them with hypothetical questions rather than, for example, engaging with the digital transformation of potlach as a contemporary practice and recently reflected in the launch of a virtual tour of the U’mista Cultural Centre in the ‘Namgis First Nation on Alert Bay, Vancouver Island’ (see CBC, 2020). Indeed, contemporary indigenous peoples have ‘rarely [been] included in academic or popular accounts on digital media’ (Coleman, 2010: 490; see also Ginsburg, 2008). When considered, their engagements with the digital are often primarily, and undoubtedly importantly, discussed with regards to experiences of diaspora such as Howard and Rensel’s (2004) work with the Rotuma, whose ancestral home lies ‘near’ the Fijian mainland, or with regards to the crafting of digital cultural databases (e.g. Strathman, 2019). Missing are broader contemporary ethnographic engagements with indigenous peoples to address more universalizing social theories of platformization, possibly due to what Ginsburg (2008) describes as a trend in digital studies at large:

[The] techno-imaginary universe of digital eras and divides. . . has the effect of reinscribing onto the world a kind of “allochronic chronopolitics”. . . in which “the other” exists in a time not contemporary with our own. Platforms, thus, re-stratify the world along the lines of late modernity, despite the utopian promises by the digerati of the possibilities of a 21st-century McLuhanesque global village (pp. 130–131).

By not engaging with lived indigenous experiences with platforms, proponents of participatory, gift-oriented perspectives have had little to respond to critiques focused on the intrinsic capitalist entanglement of platforms. If anything, the gift or participatory aspects of platforms has been argued to have become ‘reframed as valuable market strategy’ (Fourcade and Kluttz, 2020: 4) by platforms, with platforms readily monetizing, for example, the gifts of free codes provided by their users (Fourcade and Kluttz, 2020).

Our project does not set out to make any universalizing arguments about the participatory potentials of platforms. Still, we suggest that theorizations surrounding platform capitalism, and thus about the ‘nature of platforms’ – what they are and how they, by their design, transform the world – have yet to withstand the challenge of an anthropological ethnography of a gift economy in the digital age, or more broadly of an anthropological engagement with platforms that looks beyond the self-imposed parameters of Platform Studies. As Graeber (2012) has so eloquently noted,

for at least a century, anthropologists have largely played the role of gadflies: whenever some ambitious European or American theorist appears to make some grandiose generalizations about how human beings go about organizing political economic, or family life, it’s always the anthropologist who shows up to point out that there are people. . . who do things exactly the other way around (p. 393).

Margaret Mead’s research in Papua New Guinea and Samoa, Oceanic neighbours of Solomon Islands, is, at least popularly, among the most well known case for anthropological disruptions to universalising theories. Based on her Oceanic fieldwork, she established, for example, the social construction of gender and childrearing, fuelling the 1960s sexual revolution in the ‘West’ and challenging generalized yet US-centric discourses about family dynamics (King, 2019). We contend that Solomon Islands holds a similar potential for anthropologists to play the vexatious role of the gadfly, in this case, of Platform Studies. On the one hand, as described earlier, Solomon Islands is one of the last places to have widespread access to digital technologies and the Internet offering a unique and timely perspective on digital adoption and adaptation. On the other hand, a focus on Solomon Islands positions our research uniquely to decentre both the so-called Western and Eastern perspectives that have dominated the agenda of digital studies so far (see Shome, 2019), simply because of the persistence of *other* economic modes of production, distribution and consumption as well as associated values, the dominance of horticulturalism over capitalism and of reciprocal exchange over individual accumulations of wealth in everyday life.

Anthropologists have long recognized that there are many ‘unimagined’ users beyond the ‘average’ or ‘imagined’ user which are usually white men (Coleman, 2010). They have increasingly engaged with the many ‘small but necessary details that render the materiality of media (and hence its particular affordances and constraints) not only heterogeneous but fully cultural, social, and above all, political’ (Coleman, 2010: 491). Still, they have rarely engaged in direct dialogue with interdisciplinary platform studies and their current engagement based on a definition that is, in de Kloet et al.’s (2019) words ‘institutional’ and ‘needs to be opened to the realms of the social and the cultural’ (p. 249); and we contend, also to the ‘cultural’ beyond the textualized definitions of culture at the heart of primary methodological approaches in Platform Studies today – discourse, textual, content and network analyses.

While textuality is part of culture, it is not culture in and of itself. By focusing primarily on culture as text, either literally or metaphorically as something that the researcher ‘reads over the shoulders of people’, the embodied experience of everyday life is completely missed. As Varisco (2018) argues, ‘it is the face-to-face encounter that informs the process of ethnography, not a passive reading over or into the behaviour observed. Ethnography is an eye-to-eye endeavour informed through dialogue, not over- the- shoulder guesswork’ (p. 41). While we argue that such a cleavage between praxis and language is present in all societies, it is most acute in societies that are not, for most of the population, text-based – the most extensive study on literacy in the country suggests that only 17% of the population are literate (Asian South Pacific Bureau of Adult Education, 2007) – and that are not, for most of the population, deeply embedded in capitalist economic activities. Looking at platforms from this perspective, one that is ethnographic, non-text centric and non-capitalist, casts a unique and provocative, take on a phenomenon radically reshaping social relationships across the world today. We do so in the following with a focus on the platformization of Solomon Islands economy and consider how this platformization links to non- or pre-platformized practices, specifically the significance of ‘informal’ bush markets in everyday life.

## Platformizing bush markets

### The problem of availability

In the ‘West,’ classified sections of newspapers, garage sales, flea markets and their digital analogues often represented a second economy in complement and opposition to brick and mortar or catalogue-based retail or their translations into digital retail platforms such as Amazon. The situation in Solomon Islands is different. Beyond small canteens that usually only sell simple products such as rice and canned tuna, formal retail businesses are primarily located in Honiara or other (peri-)urban areas of the country. For the vast majority of islanders, ‘informal’ markets^1^ represent the main means to access foods and goods instead.

Markets are central to everyday life and livelihoods. While urban retail shops largely trade in imports, markets, from Honiara’s Central Market to the many bush markets in rural areas, provide access to Solomon Islands grown and caught foods, and other goods necessary for social and cultural reproduction such as shell valuables or dolphin teeth. Historically and today, these markets have, especially in Malaita, played ‘an analogous structural-functional role’ (Ross, 1978: 119) to, among others, the circulatory exchange systems of the aforementioned Kula Ring, ‘[delivering] the benefits of economic specialization and areal sociocultural integration’ (Ross, 1978: 119). They have, for example, allowed the Langalanga-speakers to specialize in the production of shell money, which they, in turn, exchange with other saltwater peoples such as the Lau-speakers for raw sells or with bush peoples such as the Baegu-speakers for foods such as yams and taro (Ross, 1978); and they do, whenever they take place, provide an opportunity for ‘people of different cultural backgrounds. . . and moral understandings [to] interact’ (Busse and Sharp, 2019: 127).

Especially goods with high significance for social and cultural reproduction such as shell valuables, are, however, comparatively rare, at least at rural markets that do not immediately border on settlements that manufacture them; and they are often considered overprized at the urban markets that sell them to better paid bureaucrats as well as the occasional tourists. Instead, despite their centrality for bridewealth exchanges, compensation payments during reconciliation processes, and mortuary exchanges, and thus, to core rituals that hold together the fabric of Solomon Islands societies, that solidify claims to leadership as well as belonging to particular language groups, including the right to live in their territories, these goods are often solely accessible and affordable through word of mouth. In other words, someone searching for items that are central to processes of social reproduction relies on their immediate social network to identify individuals who are from individual sellers who for one reason or another are looking to sell or exchange animals and objects such as pigs and piglets or shell money.

Both word of mouth and bush markets have historically challenged the availability of goods based on both the sellers and possible customer’s mobility, typically reducing one’s ability to exchange and sell goods to one’s immediate vicinity. Consider the case of our main field site, Gwou’ulu village. Located on the northern border of the Lau Lagoon, villagers maintain trade relations up and down the coast, with the To’abaita to the West and other Lau villages to the South. The village also maintains relations with the inland, or bush, Baelelea connected overland by a road and network of trails. They trade with each other at weekly markets in what our predecessor in the lagoon, Maranda (2002) called a ‘land-sea synergy’ (see also Ross, 1978) wherein fish is exchanged for garden produce and garden produce is exchanged for fish.

Stephanie would regularly join women to these markets. They would paddle a dugout canoe, navigating the often hostile waves for at least 2 hours, depending on the tides. Through a mangrove swamp that loomed overhead like a tunnel, they would emerge at a beachhead, tie down their canoe and then climb up a small but treacherous slope to a bush market, rows of wood tables under small thatch roofs. The problem with this system comes down to the findability of goods and services. Every day at a bush market is unique in the products that are for sale. Stephanie could never be sure what she would find. Her group would often get lists from other women in the village asking to bring particular goods – if they were available. They would rush from seller to seller hoping to find the particular item we had been instructed to bring, and to do so before someone else snatched them away from them. Rarely were they able to locate everything, occasionally resulting in a frantic search by the person requesting the item, and we recorded a fair share of instances when bridewealth payments were postponed simply because the husband’s family was unable to procure all the agreed upon items.

We ran into this problem with findability several times ourselves. For example, it took us weeks before we were able to buy a piglet. We had to mobilize all of our networks which in turn reached out to their relatives in the neighbourhood to identify a pregnant sow with as of yet unreserved piglets. When we found a piglet, it was based a half-day trip away and we had to wait to pick it up until more reasons had accumulated for such a trip, risking losing the promised piglet again to another buyer. Facebook offers a digital solution to this problem with findability and Solomon Islanders have quickly found ways to use the platform to mediate this very problem. They have started and actively participate in Facebook groups such as ‘Buy and Sell in Solomon Islands’ (over 45,300 members, November 21, 2020),^2^ ‘Buy and Sell in Honiara (Solomon Islands)’ (over 57,700 members, November 21, 2020) or ‘For Sale and Wanted in Honiara’ (3700 members, November 18, 2020).

### Digital findabilities

The use of media platforms to facilitate exchanges of goods and services has a long history on the Internet, the most famous example in the ‘West’ being the website Craigslist. A digital version of a newspaper classified advertisements section. This website was started by Craig Newark in 1995 as an email list, evolving into a website in 1996 where people advertise goods and services they wish to buy, sell or even exchange. While Craigslist boasts over 20 billion site views a month and covers over 700 cities and regions, Solomon Islands is not among them. No matter, Facebook Groups work just as well for Solomon Islanders, and the examples for items sold and bought there are vast.

**Figure 1.**
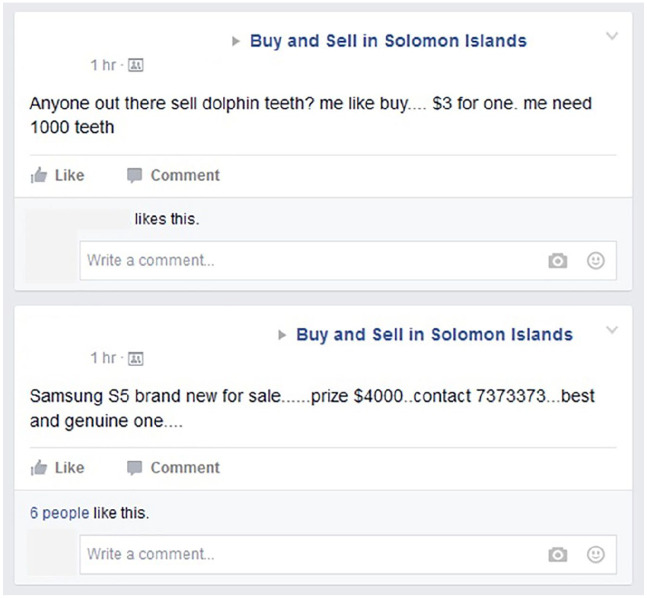
Buy and Sell in Solomon Islands, August 18, 2015.

The screenshot from the Buy and Sell page depicted in Figure 1 is indicative of this vastness. It demonstrates at once the scope but also the general economics of Solomon Islands itself. Here, one man asks if anyone is selling dolphin teeth. A sharp costumer. Dolphin teeth are, similar to the aforementioned shell money, used as a traditional form of currency. Then, immediately next, another man offers something quite different, a Samsung S5 smartphone. Also, pre-made meals are advertised, often with pictures that remind of the market stalls selling the same produce throughout Honiara. One post, for example reads,

Bonbon fish and casava/ banana on sale again today and everyday till Fridays. . . place ur order and get your fill. . . we can also delivery for you. . . on $30 and $40 nmoa.. fish and casava, or fish and banana. .. at THE KITCHEN!! (Optimal Buy & Sell – Solomon Islands, October 7, 2020)

Solomon Islanders also sell airline tickets this way as well as mobile phones, cars, auto parts, fibreglass boats, soaps and toys and more local goods from birthday cakes to pigs.

So what does all of this mean for how Solomon Islanders sell and obtain goods on an everyday basis as well as for ritually significant events? First and foremost, it reduces the need both for uncertain trips to bush markets and for word of mouth. Items have simply become more findable, with these Facebook groups not necessarily replacing but at least complementing non-Internet based searches for goods. As Lipset (2013) notes for the Murik of neighbouring Papua New Guinea, the telephonic function of mobile phones has had a similar effect. With the proliferation of mobile phones, it suddenly became possible to, for example, ‘call kin at trade-stores to tell them to buy things’ (p. 341). What telephony has not allowed for is to extend trading beyond existing social networks, instead it has merely extended the capacity to exchange information with more distant members of the network. Facebook-based Buy and Sell groups offer a much wider selection of goods, not that different from bush markets to, in essence, find goods that cannot be procured from immediate trading partners.

Most Buy and Sell groups mainly advertise products that are based in Honiara, but that is not a problem per se. For example, when we were looking for our piglet, some also reached out to contacts in Honiara. The Lau regularly travel back and forth between Honiara and the Lau Lagoon, with people arriving from town on a weekly basis. If one has the money to buy a product and to pay for the transport cost obtaining it from Honiara is usually not a problem. It is often even easier than getting it, for instance, from bush-based relatives as our piglet, simply because someone is bound to make a trip within a relatively short time.

### Non-capitalist buy and sell groups

While increasing the findability of goods, Buy and Sell groups are much more like bush markets than the retail businesses in towns. In other words, Facebooks markets do not fundamentally disrupt dominant trading patterns. For example, Buy and Sell groups provide for the same kind of flexibility as bush markets or word of mouth trading within existing social networks. A person can offer a good whenever they desire to. They do not have to be regularly ‘open’, and a person does not have to have ‘stocks’ or ‘inventory’ like the urban retail businesses. They also do not require ‘employers’ and ‘employees’. Instead, anyone with a Facebook account can both buy and sell on this online market. Additionally, Buy and Sell groups effectively circumvent any government attempt to tax businesses, just like most market stalls do, while by no means constituting a secondary source of cash income. As already noted ‘informal’ markets, be they offline or online, are how most Solomon Islanders access the cash economy, often as a mere secondary activity alongside subsistence agriculture and fishing (see Sharp and Busse, 2019).

Thus, Buy and Sell groups, in no way disrupt dominant non-capitalist economic patterns in Solomon Islands. Nor could it be argued that these groups further Solomon Islanders’ integration into the global capitalist economy. On the contrary, if anything they provide opportunities for Solomon Islanders to circumvent the usually foreigner-run, retail businesses in Honiara. Solomon Islanders may need them, or similar businesses abroad, to source some of the goods that are sold on ‘informal’ markets. However, the moment these goods are acquired and enter offline and online markets they enter systems of exchange that predate the arrival of capitalism in the country. These systems are not about accumulating wealth in the capitalist sense. Instead, they are about everyday livelihoods and about accumulating wealth in the Melanesian sense, wealth that is relational or dividual, ‘where the creation and affirmation of relationships is the key goal of interaction, [and where] exchange is carried out precisely in order to foster mutual recognition’ (Robbins, 2008: 48; see also Sharp and Busse, 2019).

The women that Stephanie accompanied to bush markets often varied. Largely those women, or in extension families, that had an immediate need for cash or particular bush produce went to markets. If they had no such needs, but still some ‘spare’ fish or other produce, they would rather share them within their social and primarily kin networks than selling them at these markets. Sharing foods and goods, at times even when it meant not immediately having enough oneself, is central to Lau socialities. As Lau ethnomusicologist Hundleby (2017) notes, acts of giving and receiving are ‘the essential foundations upon which trust is built’ (p. 110) and define what makes a ‘good’ person; and in turn, those who do not share risk social exclusion, even the right to reside in the villages that are situated on communally, customarily owned land (see McDougall, 2017).

By operating like bush markets, Buy and Sell groups comparatively easily allow for replicating this system, much more so than, for example, even indigenous run small-medium scale ‘brick and mortar’ businesses. Research on indigenous retail businesses in the Pacific has found that it is largely family-run businesses that are deeply embedded in their communities and that share their financial ‘profits’ with these communities that tend to succeed (e.g. see Curry, 2005; Scheyvens et al., 2020). Simultaneously, this research has highlighted that these businesses often necessarily struggle with survival since they do have to find ways to negotiate the needs for inventory, savings and labour with demands for reciprocal giving (Curry, 2005). Facebook’s Buy and Sell groups do not contain these challenges, at least not to the same degree, and are, thus, flourishing as digital extensions of bush rather than capitalist markets.

Crucially and finally, this is also the case because even the foreign goods that are sold and obtained through Buy and Sell groups are often acquired for social reproduction. A vast majority of foreign goods sold on these groups are mobile phones, computers and accessories for them. As we have argued elsewhere (e.g. Hobbis, 2020; Hobbis and Hobbis, 2020), digital technologies and media are deeply entangled with processes of moral social reproduction through reciprocal exchange. For example, mobile phones primarily serve as ‘kinship technologies’ (Hobbis, 2020). They are valued because they allow their users to more easily negotiate reciprocal relationships across urban-rural divides, among others, by providing the technological means for coordinating participation in ritually significant events such as mortuary rites among kin networks spread across the country (Hobbis, 2020). By facilitating the sale of mobile phones and other digital technologies, Buy and Sell groups essentially extend their availability and, thus, further strengthen non-capitalist exchanges rather than elevating the presence of (platform) capitalism in Solomon Islands Facebook users’ lives.

## Facebook capitalism in a relational economy

On November 17th, 2020, the Solomon Islands Government announced that they are preparing to ban Facebook. Similar to the government of neighbouring Papua New Guinea (PNG) (see Roy, 2018), they expressed severe concerns about the information that is shared on Solomon Island Facebook groups. In PNG, the official concern was over the popular distribution of pornographic images, driven as part of a campaign for a more moral Internet. In Solomon Islands, the government echoed concerns over harmful content, especially deleterious affects it may have on younger users, while citing a desire to prohibit critical and abusive language directed towards government ministers including ‘character assassination and defamation’ (Wickham and Doherty, 2020).

Videos and images depicting sex and violence are standard on some pages as are verbal insults which are, especially in Malaita, historically and today, one of the primary reasons for outbreaks of violence between individuals and, usually, in extension their kin networks (see Ross, 1978). This content, however, is often left up even if it is reported to Facebook’s content moderators. In other words, while Facebook is, among others, actively removing groups that support Netherlands ‘Black Pete’ in an attempt to curb down on ‘hate speech’, Facebook has, to the best of our and our Solomon Islander interlocutors’ knowledge, not shown any interest in helping them respond to their concerns about online ‘hate speech’. This is true for politically contentious issues such as discussions about corruption to more broadly agreed on concerns about what they deem to be sexually inappropriate contents.

As Srnicek (2017) argues, platforms survive by ensuring that no competitor does better than them, so they try to make sure that they are the only option for a particular service in all corners of the globe. This is not much different from how colonialism was driven by competition between European powers, including the need to bring as many areas of the world into one’s jurisdiction to beat out other imperial interests. As previously noted, Solomon Islands was one of the last places to be colonized by European powers with Britain primarily establishing a protectorate there because they did not want France to do so, rather than because they saw value, as they defined it, in the archipelago. Indeed, when Solomon Islands gained independence ‘the British [saw] the process as one of their gaining independence from the Pacific rather than the territories like the Solomons winning independence from them’ (Bennett, 1987: 321). While the reasons for this are manifold, one of the most important concerns the capitalist exploitability of the Solomons: there simply did not seem to be enough desired natural resources to warrant the costs and efforts of colonization.

The presence of Facebook in Solomon Islands is comparable. Facebook is available in Solomon Islands because Facebook seeks to dominate all possible markets as exemplified in their Internet.org initiative which offers limited free access to some platforms on the Internet, including Facebook, in emerging markets. Simultaneously, the value of Solomon Islands capitalist market for Facebook is minimal at best, and so it seems, not worth the investments necessary to invest resources into the country. Solomon Islands, at the very margins of global capital, is of some interest to Facebook but not enough for Facebook to honestly care about what is going on in this section of its platform. In other words, the relationship between platforms and Solomon Islands is fundamentally different from, for example, the relationship between platforms and Saudia Arabia: While Khalil and Zayani (2021) observed ‘a symbiotic relationship of mutual accommodation’ (p. 212) between Netflix and the government of Saudi Arabia, with Netflix budging on freedom of speech in exchange for market access, Facebook (or Netflix for that matter – it is unavailable in Solomon Islands) have shown no public interest in any form of accommodation to better, and reliably, access Solomon Islands (potential) customer base.

There is no evidence that Facebook cares that Solomon Islanders regularly do not even use the marketplace function of Facebook for its Buy and Sell groups and that Facebook is, therefore, less able to collect data on Solomon Islanders and their purchasing decisions. Facebook is also not able to reliably quantify, and, thus, extract sellable data, from many of its Solomon Islander users in other ways. Most Solomon Islanders we know on Facebook have multiple accounts, they perpetually delete accounts, forget passwords, or even operate multiple accounts to reflect what they need these accounts for. Just like Solomon Islanders have multiple SIM cards (Hobbis, 2017), they have multiple Facebook accounts so that they can flirt outside of marriage through one account, engage in Buy and Sell activities on another, express possibly politically contentious issues on yet another and so on. Solomon Islanders regularly do not use their proper names for Facebook accounts, and sometimes not even pictures of themselves, if they depict any human images at all. Solomon Islanders also rarely provide any kind of true information about themselves on Facebook, from their age, to their location of residence, to where they went to school. Individual accounts are, thus, by no means attached to individual identities.

This obscurity has many reasons, some practical, some more deeply embedded in the particular cultural context of the place. Disclosing one’s true name can, for example, render oneself susceptible to witchcraft and, hence, many Solomon Islanders carefully guard their identities when engaging with digital technologies (Hobbis and Hobbis, 2017). Algorithms, thus, struggle to quantify Solomon Islands Facebook users. And why should they? There are also no substantial businesses that may even be interested in advertising to Solomon Islanders through algorithmic, suggested posts online, excluding perhaps organizations such as the UN and their various campaigns – though we have yet to find evidence for this. Instead, most retail businesses run free Facebook pages that they use to advertise their services, while everyone else using Facebook for ‘business’ does so through Buy and Sell groups.

## Conclusion

What does all of this mean? Most simply, it means that the story of platformization in Solomon Islands is not, at least not primarily, about platform capitalism. Despite de Kloet et al.’s (2019) proposition that platforms ‘commodify’, in the capitalist sense, ‘all social relations by collecting, algorithmically processing, circulating and selling user data’ (p. 249), this is only marginally true for Solomon Islands. While Facebook is collecting such data about Solomon Islanders, the way Solomon Islanders use Facebook and primary economic relations in Solomon Islands, limit both the ways in which such data can be collected and possible customers for such data. Instead, Solomon Islanders use the platform that Facebook provides to realize their own vision of the world that addresses but does not centre on a capitalist-, commodity-centric worldview. Facebook has even offered Solomon Islanders an opportunity to continue, revive and adjust moral economic practices that have been endangered by retail capitalism. By providing an online extension of informal bush markets, Solomon Islanders are able to use Buy and Sell groups effectively to engage in trade for purposes of social reproduction, to acquire otherwise difficult to find ritually significant objects such as shell valuables, as much as the digital technologies that have become central to the maintenance of reciprocal relationships across urban-rural distances.

Platformization is, thus, here a story of moral social reproduction that is tied to a primarily horticulturalist rather than capitalist economy. This defies claims that the ‘tensions between agency and architecture’ (Plantin et al., 2018: 297) constitute ‘inter-capitalist struggles’ (Prey, 2020: 8) and that studying platforms only makes sense if it is done from the perspective of their designers, the platform itself. Instead, our research suggests a need for decentring Platform Studies. There is more to platforms and, thus, more to Platform Studies than platform capitalism. We contend that to understand how platforms are transforming the world at large, beyond the ‘sets of relations’ (van Dijck, 2013: 26) that are performed surrounding and through platforms at capitalist cores, it is crucial to also pay attention to how primarily non-capitalist, non-industrial economies are interfacing with platforms. This means moving beyond the ‘average user’ within capitalist relations, to move beyond ‘the much bigger, invisible technical dimensions of the platforms’ (Nieborg and Helmond, 2019: 198) and return to the diverse user through a more holistic, ethnographic engagement with diverse economic social relations. This opens up new horizons in platform research, in our case, what may be called, ‘platform horticulturalism’ and what in other instances may be anticipated as ‘platform hunter-gathering’, ‘platform pastoralism’ and ‘platform agriculturalism’.

## Notes

1. By ‘informal’ or ‘bush markets’ we are by no means referring to the ‘market *as a principle*’, as ‘abstract ideas about a mode of exchange involving money and organized in accordance with supply, demand and price’ (Busse and Sharp, 2019: 126). Instead, following Busse and Sharp (2019), we focus on ‘market *places—*gatherings at particular places focused on trade’ (p. 126), be they offline or online, and we consider market places as being necessarily about both the social and the economic.

2. The name of this page is regularly updated, it has, for example, been changed to ‘Optimal buy & sell – Solomon Islands’.
